# Wine of Cascara Sagrada

**Published:** 1898-09

**Authors:** 


WINE OF CASCARA SAGRADA. A gentle laxative and tonic wine suitable for dogs and other small pet animals, may be made by macerating one part of fluid extract of cascara sagrada in eight parts of sherry wine. After macerating for eight days and filtering, the mixture is ready for use.

